# Risk Assessment for Preeclampsia in the Preconception Period Based on Maternal Clinical History via Machine Learning Methods

**DOI:** 10.3390/jcm14010155

**Published:** 2024-12-30

**Authors:** Yeliz Kaya, Zafer Bütün, Özer Çelik, Ece Akça Salik, Tuğba Tahta

**Affiliations:** 1 Department of Gynecology and Obstetrics Nursing, Faculty of Health Sciences, Eskişehir Osmangazi University, Eskişehir 26040, Türkiye; 2 Hoşnudiye Mah. Ayşen Sokak Dorya Rezidans, A Blok no:28/77, Eskişehir 26130, Türkiye; zaferbutun@hotmail.com; 3 Department of Mathematics—Computer Science, Faculty of Science, Eskisehir Osmangazi University, Eskisehir 26040, Türkiye; ozer@ogu.edu.tr; 4 Department of Gynecology and Obstetrics, Eskisehir City Hospital, Eskişehir 26080, Türkiye; eceakca8@gmail.com; 5 Health Services Vocational School, Ankara Medipol University, Ankara 06050, Türkiye; tahtatugba@gmail.com

**Keywords:** preeclampsia, preconception, pregnant, artificial intelligence, machine learning

## Abstract

**Objective**: This study was aimed to identify the most effective machine learning (ML) algorithm for predicting preeclampsia based on sociodemographic and obstetric factors during the preconception period. **Methods**: Data from pregnant women admitted to the obstetric clinic during their first trimester were analyzed, focusing on maternal age, body mass index (BMI), smoking status, history of diabetes mellitus, gestational diabetes mellitus, and mean arterial pressure. The women were grouped by whether they had a preeclampsia diagnosis and by whether they had one or two live births. Predictive models were then developed using five commonly applied ML algorithms. **Results**: The study included 100 mothers divided into four groups: 22 nulliparous mothers with preeclampsia, 25 nulliparous mothers without preeclampsia, 28 parous mothers with preeclampsia, and 25 parous mothers without preeclampsia. Analysis showed that maternal BMI and family history of diabetes mellitus were the most significant predictive variables. Among the predictive models, the extreme gradient boosting (XGB) classifier demonstrated the highest accuracy, achieving 70% and 72.7% in the respective groups. **Conclusions**: A predictive model utilizing an ML algorithm based on maternal sociodemographic data and obstetric history could serve as an early detection tool for preeclampsia.

## 1. Introduction

Preeclampsia (PE) is a hypertensive disorder of pregnancy that poses serious risks to both maternal and fetal health. PE can lead to maternal death during pregnancy and may also result in long-term health complications, such as chronic hypertension, in the mother after delivery [1]. The early detection of PE is crucial not only to mitigate its immediate impacts during pregnancy but also to reduce its long-term effects. PE is a condition uniquely diagnosed during pregnancy, as per the American College of Obstetricians and Gynecologists (ACOG) criteria. It is characterized by new-onset hypertension (blood pressure ≥140/90 mm Hg) after 20 weeks of gestation, accompanied by proteinuria (≥300 mg in a 24 h urine collection) or, in the absence of proteinuria, other signs of organ dysfunction, such as thrombocytopenia, renal insufficiency, impaired liver function, pulmonary edema, or cerebral or visual disturbances [1]. These diagnostic criteria underscore that preeclampsia is identified only when these clinical signs emerge during pregnancy, thus limiting opportunities for early preventive interventions. This timing restriction highlights a significant clinical gap, as identifying preeclampsia risk before conception could allow for earlier management strategies, potentially reducing the incidence and severity of this condition. Previous studies have explored various factors, including maternal characteristics, obstetric and medical history, ultrasound measurements, genetic markers, and both basic and advanced biomarkers, to develop predictive models for PE. These factors have contributed to the creation of nomograms that aim to predict PE during pregnancy. However, the majority of existing research has focused on data collected during pregnancy, with limited investigation into the potential of preconception data. Currently, the lack of reliable screening tests to identify preeclampsia risk in early pregnancy, combined with a shortage of evidence-based preventive strategies, limits the ability to manage preeclampsia effectively.

Artificial intelligence has garnered significant interest from researchers and the biomedical industry due to its capacity to analyze vast datasets, deliver precise outcomes, and optimize processes for enhanced results. Like in many other fields, AI is extensively applied in healthcare for predictive modeling, diagnosis, early detection, and patient monitoring. Machine learning (ML) algorithms have been employed to analyze the complex relationships between preconception variables and the risk of developing PE. The use of such advanced algorithms allows for more accurate predictions by identifying subtle patterns and interactions within the data that traditional methods might overlook. ML models based on preconception data could be valuable tools for the early prediction of preeclampsia. Such an approach has the potential to improve maternal and fetal health outcomes by enabling earlier detection and intervention, thus reducing the incidence and severity of preeclampsia-related complications.

The aim of this study was to develop an ML model for predicting preeclampsia using maternal variables obtained in the preconception period. The utilization of preconception data presents a promising opportunity for the early identification of women at risk for PE, potentially enabling early intervention and preventive strategies before pregnancy begins.

## 2. Materials and Methods

After obtaining ethical approval, we retrospectively collected maternal and pregnancy-related data from patients admitted to the Obstetric Clinic at Eskisehir City Hospital between 1 January 2022 and 31 December 2023. We identified the inclusion criteria as follows:

- Having hospital admittance in the first trimester of pregnancy;
- Being > 18 and <40 years old;
- Having a spontaneous, non-anomalous, and singleton pregnancy;
- Having all sociodemographic data for the preconception period (maternal age, body mass index (BMI), smoking habit, history of diabetes mellitus, history of gestational diabetes mellitus, mean arterial pressure, and whether PE was experienced previously) and obstetrics history (gravida, parity) available;
- Having a documented pregnancy outcome with preeclampsia status (presence or absence) known.

Data on maternal age; body mass index (BMI); gravidity; parity; smoking status; history of diabetes mellitus, gestational diabetes mellitus (GDM), or hypertension; mean arterial pressure; and previous history of preeclampsia were collected retrospectively from electronic hospital records. The study population consisted of pregnant women who had attended antenatal clinics, with data gathered from a comprehensive hospital database to ensure reliability and completeness. In line with the American College of Obstetricians and Gynecologists (ACOG) criteria [2], patients were grouped based on the presence or absence of preeclampsia (PE) during pregnancy. Participants were classified based on their obstetric history as either nulliparous or parous. Parous refers to pregnant women who have a history of one or more births, including primiparous women (who have given birth once) and multiparous women (who have given birth two or more times). For further stratification, participants were also classified into subgroups as nulliparous (pregnant women who had never given birth before the current pregnancy) and parous (pregnant women who had previous live births) depending on the number of live births to refine the predictive modeling process by parity. Stillbirths were not included in the classification criteria.

Data preprocessing steps included addressing missing values, normalizing continuous variables, and encoding categorical variables where applicable. Since electronic health records may contain incomplete information, missing values were managed through different imputation methods: mean imputation for continuous variables, mode imputation for categorical variables, and model-based imputation where complex interactions were identified. Additionally, continuous variables such as BMI and mean arterial pressure were normalized to ensure consistency across the dataset, enhancing model performance and comparability. Data for both nulliparous and parous women were reviewed separately and used for separate model-building processes, allowing for tailored models that took into account differences in pregnancy history and related factors.

To develop robust predictive models for identifying preeclampsia risk, several machine learning (ML) algorithms were employed, including the extra trees classifier, light gradient boosting machine (LGBM) classifier, extreme gradient boosting (XGB) classifier, logistic regression, and random forest classifier. Each algorithm was selected based on its strengths in handling complex, multidimensional healthcare data. These models were trained on 80% of the dataset, while the remaining 20% was reserved for testing and model evaluation. The selection of these algorithms was aimed to balance interpretability with predictive accuracy. Ensemble methods such as extra trees, LGBM, and random forest were chosen for their ability to capture complex patterns in the data, while logistic regression was included for its simplicity and ease of interpretation in clinical applications.

The training process was conducted using an 80/20 train–test split, a common method to prevent overfitting and assess the model’s generalizability by building models on a substantial portion of the data while preserving a separate test subset for unbiased evaluation. To further validate the model’s robustness, k-fold cross-validation (with k = 10) was applied, iteratively testing model performance on various data folds by splitting the dataset into 10 equal parts, where each fold served as a test set while the remaining folds were used for training. Model success rates were evaluated based on accuracy, sensitivity, and specificity, as derived from confusion matrix metrics. Sensitivity was particularly emphasized as a key metric to assess the model’s ability to identify patients at risk for preeclampsia. Additionally, the area under the receiver operating characteristic (ROC) curve (AUC) was calculated to assess the model’s discriminative power, as it measures the trade-off between sensitivity and specificity, providing a more comprehensive evaluation of the model’s overall performance. Confusion matrices provided insights into classification accuracy by comparing predicted and actual classifications, which were essential for identifying false positives and negatives. This rigorous evaluation process supported the selection of the most suitable model for predicting preeclampsia risk among nulliparous and parous women. Minimizing false positives is crucial to avoid unnecessary interventions that could cause undue stress or medical costs, while reducing false negatives ensures that high-risk patients are correctly identified and receive timely preventive care, which is essential for improving maternal and fetal outcomes.

**Figure 1:**
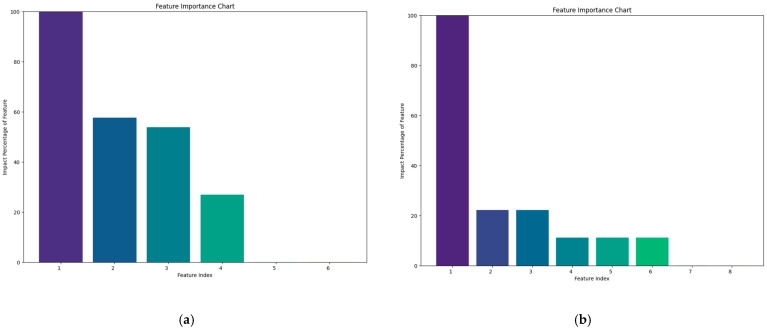
(**a**) Feature importance chart of the variables for the nulliparous mother group. 1: Body mass index, 2: gravida, 3: maternal age, 4: history of hypertension. (**b**) Feature importance chart of the variables for the parous mother group. 1: History of gestational diabetes mellitus, 2: body mass index, 3: history of hypertension, 4: smoking, 5: history of diabetes mellitus, 6: history of preeclampsia.

To identify significant predictors of preeclampsia, the permutation feature importance method was employed. This model-agnostic technique quantifies the importance of each variable by measuring the drop in model accuracy when the values of that variable are randomly shuffled. The magnitude of the accuracy drop indicates how much the model’s performance depends on that specific variable, adding to the method’s versatility and interpretability. The resulting permutation feature importance plots (Figure 1a,b) illustrate the relative impacts of factors such as maternal age, BMI, mean arterial pressure, and history of hypertension on preeclampsia risk. This approach provided a transparent and interpretable way to rank predictors, aiding in understanding the most influential factors in the model.

Statistical analyses were conducted to assess differences between groups and validate findings. The Shapiro–Wilk test was utilized to evaluate the normality of continuous variables. Data are presented as mean ± standard deviation for normally distributed continuous variables and as median (minimum–maximum) for non-normally distributed variables. For comparisons between groups, independent samples *t*-tests were used for normally distributed data, while the Mann–Whitney U test was applied for non-normally distributed data. Categorical data were compared using the chi-squared test. A two-sided *p*-value of <0.05 was considered statistically significant, indicating a robust level of confidence in the observed associations.

## 3. Results

Table 1 shows demographic and obstetric information for the 100 mothers included in the study. These patients were classified as those who were diagnosed with PE and those who were not. Then, all mothers were classified as nulliparous and parous. Overall, 22 and 25 nulliparous mothers experienced PE and did not, respectively, whereas for parous mothers, 28 and 25 experienced PE and did not, respectively.

**Figure 2:**
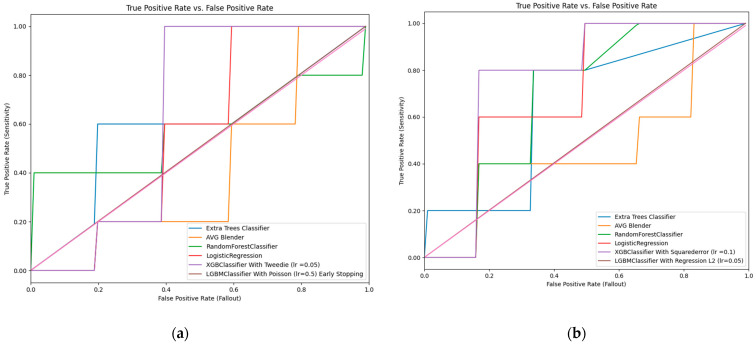
(**a**) Receiver operating characteristic curve graphs of the nulliparous mothers’ variables. (**b**) Receiver operating characteristic curve graphs of the parous mothers’ variables.

In the first part of this study, the nulliparous mothers who experienced and did not experience PE were analyzed statistically. While only BMI during the preconception period exhibited statistically significant differences between the two groups (*p* = 0.003), as seen in Table 1, BMI, gravida, maternal age, and the history of hypertension were the feature importance parameters (Figure 1a). After training the dataset, the ML algorithms were applied to the test data. The XGB classifier model emerged as the most effective algorithm, achieving the highest accuracy (70%) and AUC-ROC value (64%) for predicting preeclampsia based on maternal sociodemographic and obstetric history (see Table 2, Figure 2a). This model demonstrated a sensitivity of 80% and a specificity of 60% (refer to Table 3).

In the second part, the parous mothers who experienced and did not experience PE were analyzed statistically. Age, history of hypertension, and history gestational diabetes mellitus exhibited statistically significant differences between the two groups (*p* = 0.022, 0.014, and 0.001, respectively), as seen in Table 1. The history of gestational diabetes mellitus, BMI, history of hypertension, smoking habits, history of diabetes mellitus, and previous PE were the feature importance parameters (Figure 1b). Once again, the XGB classifier model proved to be the most powerful among the seven algorithms when applied to the test data after training. This model achieved the highest accuracy rate (72.7%) and AUC-ROC value (76.7%) for predicting preeclampsia based on maternal sociodemographic and obstetric history (see Table 2, Figure 2b). It demonstrated a sensitivity of 60% and a specificity of 83.3% (refer to Table 3).

## 4. Discussion

PE is a significant cause of maternal and fetal morbidity and mortality worldwide. It is among the major contributors to severe complications such as bleeding, infections, and hypertensive disorders during pregnancy, which remain critical challenges in maternal healthcare. Despite advancements in reducing maternal and fetal death rates globally, preeclampsia continues to pose substantial risks, particularly in regions with limited healthcare resources [3,4,5,6,7]. Addressing this condition through early prediction and prevention strategies is vital for improving maternal and fetal outcomes globally.

The first step to deal with this life-threatening disease is the prediction and screening of PE using identified risk factors. The Fetal Medicine Foundation (FMF) competing-risks model was developed with maternal characteristics, advanced biomarkers, and ultrasound measures of the uterine vessels [8,9]. This risk model was externally validated several times, and the trials showed significant reductions in the rate of preeclampsia, which could be evaluated as indicating the model’s effectiveness [10,11]. Tiruneh et al. [12] recently published a systemic review and meta-analysis of the externally validated prediction models of PE, and the authors screened 52 externally validated PE prediction models. Limitations of the prediction tools that use classical statistical methods are that several risk factors have been identified and the interactions between the parameters used in the nomograms are still controversial; furthermore, the ability to deal with a large number of variables is very limited [13]. To overcome these limitations, ML models are being investigated in the literature to perform the prediction of PE [14,15]. As these ML algorithms have shown better outcome performance for predicting PE than nomograms created via classical statistical methods [16], ML models are defined as possible tools for all healthcare risk prediction, including PE. However, in the limited number of reports available, the ML models did not result in a superior prediction of PE compared to statistical methods [17,18,19].

The major limitation of these ML models, regardless of the development method, is that these tools are mostly only available for high-income countries because the models require parameters such as advanced biophysical markers. As a result, it is important to establish a predictive tool based on variables that can be obtained during a basic medical examination because access to blood tests and ultrasound examinations is very limited in many parts of the world. However, it is very easy for medical workers to learn standard parameters like maternal characteristics and medical and obstetric histories. Despite this, a systematic review found that just 11 of 52 models (21.2%) used only maternal characteristics as variables, and these models exhibited similar predictive performance to that of the models using all biophysical parameters [12]. While the reports using ML models have been published more frequently recently, maternal characteristics, mean arterial pressure, uterine artery pulsatility index, and serum placental growth factor were used in almost all the reports [14,15,16]. According to our knowledge, there has been only one report that features only maternal characteristics and medical and obstetric histories to construct a predictive model for PE via ML methods in the literature [19]. Mustafa et al. recently established a model with simple and universally accessible maternal characteristics for twin pregnancies [19]. They found that the XGB algorithm yielded an AUC of 0.62 ± 0.004, but they did not interpret the other performance outcomes of the model. A notable strength of Mustafa et al.’s study [19] is its focus on universally accessible maternal characteristics, which eliminates the need for advanced biochemical or biophysical markers. This simplicity makes their approach potentially applicable in resource-limited settings. However, their focus on twin pregnancies hinders the generalizability of the findings to singleton pregnancies, which represent the majority of cases globally. In this study, we used only easily accessible parameters during the pre-pregnancy period to construct the models. In order to avoid missing data, the nulliparous and parous pregnant women were separated, and two different models were developed. The most common ML algorithms (extra trees classifier, AVG blender, light gradient boosting machine (LGBM) classifier, XGB classifier, logistic regression, and random forest classifier) were used, and we found that the XBG classifier had the best performance outcomes for the two groups, with accuracies of 70 and 72.7% for predicting PE during the pre-pregnancy period. In comparison, our study extends and distinguishes itself from the work of Mustafa et al. [19] by addressing the predictive modeling of preeclampsia through a stratified approach, separately developing models for nulliparous and parous women to account for the distinct influences of pregnancy history. Furthermore, while Mustafa et al. [19] focused predominantly on the AUC as a performance metric, our study provides a more comprehensive evaluation by incorporating additional metrics, including accuracy, sensitivity, and specificity. This multidimensional assessment offers a nuanced understanding of model performance, enhancing its clinical relevance. By employing machine learning techniques exclusively with pre-pregnancy data and universally accessible maternal characteristics, this research underscores the feasibility of predictive modeling without reliance on advanced biochemical or biophysical markers, thereby broadening the potential application of these models to resource-limited settings.

This study opens several pathways for future research aimed at improving preeclampsia prediction, management, and maternal healthcare. Further investigations integrating larger multicenter datasets could enhance model generalizability and ensure applicability across diverse populations. Future studies could also focus on incorporating additional biomarkers and genetic data into the ML models to increase predictive accuracy. Biomarkers related to inflammation, placental function, and oxidative stress, along with genetic predispositions, could refine these models and potentially aid in identifying at-risk populations even before conception. Additionally, expanding these models to incorporate real-time monitoring data from wearable devices could offer continuous risk assessment throughout pregnancy. For example, tracking parameters like blood pressure, heart rate variability, and physical activity might provide insights into evolving risk profiles, allowing healthcare providers to intervene proactively.

Another promising application of this research lies in developing a comprehensive clinical decision-support system for obstetricians. This system, enhanced with ML-based prediction tools, could assist in identifying high-risk pregnancies early on, enabling personalized care plans and reducing the likelihood of adverse outcomes. Furthermore, integrating these predictive models into telemedicine platforms could extend their benefits to remote or underserved areas, providing critical support in settings where routine prenatal screening may be limited.

Lastly, future research could focus on combining AI-based risk prediction models with public health initiatives to support preeclampsia prevention strategies. This approach could provide targeted education and resources to at-risk individuals, fostering preventive measures well in advance of pregnancy.

The limitations of this study include its retrospective design, limited dataset, and the fact that it only focused on a Turkish cohort, which may limit its broader application. Furthermore, external validation of the dataset was not performed to assess the accuracy of the predictive model.

In conclusion, although various parameters like genetic markers, advanced biomarkers, and ultrasound measurements are the most popular options for developing a predictive tool for PE, we have shown for the first time in the literature that basic variables can be sufficient to construct an ML model with a high level of performance for predicting PE in nulliparous and parous pregnancies. In future studies, researchers should focus on predicting models for pregnancy-related diseases with universally accessible maternal characteristics for low-income countries in accordance with the Sustainable Development Goals.

## Figures and Tables

**Table 1:** Demographic and obstetric findings of the patients included in the study.

		Nulliparous			Parous		
		**+**	**−**	***p***	**+**	**−**	***p***
Age (years) mean + std. dev.		27.09 ∓ 6.48	27.28 ∓ 5.26	0.856 ^b^	32.35 ∓ 5.53	29.0 ∓ 4.68	**0.022 ^a^**
BMI (kg/m^2^) mean + std. dev.		33.17 ∓ 5.28	28.79 ∓ 4.10	**0.003 ^a^**	32.25 ∓ 6.06	31.19 ∓ 4.67	0.484 ^a^
Gravida (n, %)		1.28 ∓ 0.55	1.16 ∓ 0.37	0.522 ^b^	3.04 ∓ 1.23	2.68 ∓ 0.80	0.454 ^b^
Smoking (n, %)	+	2 (9.1%)	0 (0%)	0.123 ^c^	1 (3.6%)	0 (0%)	0.340 ^c^
	−	20 (90.9%)	25 (100%)		27 (96.4%)	25 (100%)	
History of hypertension (n, %)	+	3 (13.6%)	0 (0%)	0.056 ^c^	6 (21.4%)	0 (0%)	**0.014 ^c^**
	−	19 (86.4%)	25 (100%)		22 (78.6%)	25 (100%)	
Preeclampsia (n, %)	+	Not applicable			2 (7.1%)	0 (0%)	0.173 ^c^
	−				26 (92.9%)	25 (100%)	
History of diabetes mellitus (n, %)	+	1 (4.5%)	0 (0%)	0.281 ^c^	1 (3.6%)	0 (0%)	0.340 ^c^
	−	21 (95.5%)	25 (100%)		27 (96.4%)	25 (100%)	
Gestational diabetes mellitus	+	Not applicable			10 (35.7%)	0 (0%)	**0.001 ^c^**
	−				18 (64.3%)	25 (100%)	

^a^ Student’s *t*-test, ^b^ Mann–Whitney U test (data are not normal), ^c^ chi-squared.

**Table 2:** Prognosis prediction results of different machine learning algorithms.

	Model Training Results					
	Nulliparous			Parous		
**Model Name**	**ROC-AUC**	Accuracy	Interval	ROC-AUC	Accuracy	Interval
Extra trees classifier	0.58	0.7	0.416–0.984	0.65	0.545	0.251–0.84
Random forest classifier	0.58	0.5	0.19–0.81	0.683	0.636	0.352–0.921
Logistic regression	0.56	0.5	0.19–0.81	0.7	0.636	0.352–0.921
**XGB classifier**	0.64	0.7	0.416–0.984	0.767	0.727	0.464–0.99
LGBM classifier	0.5	0.5	0.19–0.81	0.5	0.545	0.251–0.84

LGBM: light gradient boosting machine, XGB: extreme gradient boosting.

**Table 3:** Classifier confusion matrix.

	XGB Classifier					
	Nulliparous			Parous		
**Preeclampsia**	**Yes**	No	%	Yes	No	%
Yes	4	1	80.0 ^a^	3	2	60.0 ^a^
No	2	3	60.0 ^b^	1	5	83.3 ^b^

XGB: extreme gradient boosting, ^a^: sensitivity, ^b^: specificity.

## Data Availability

The datasets used and/or analyzed during the current study are available from the corresponding author upon reasonable request.

## References

[1] American College of Obstetricians and Gynecologists (2013). Hypertension in pregnancy: Report of the American College of Obstetricians and Gynecologists’ Task Force on Hypertension in Pregnancy. Obstet. Gynecol..

[2] American College of Obstetricians and Gynecologists (2020). Gestational hypertension and preeclampsia: ACOG practice bulletin, number 222. Obstet. Gynecol..

[3] World Health Organization (2024). Maternal Mortality. https://www.who.int/news-room/fact-sheets/detail/maternal-mortality.

[4] Dimitriadis E., Rolnik D.L., Zhou W., Estrada-Gutierrez G., Koga K., Francisco R.P.V., Whitehead C., Hyett J., da Silva Costa F., Nicolaides K. (2023). Pre-eclampsia. Nat. Rev. Dis. Primers.

[5] Magee L.A., Brown M.A., Hall D.R., Gupte S., Hennessy A., Karumanchi S.A., Kenny L.C., McCarthy F., Myers J., Poon L.C. (2022). The 2021 International Society for the Study of Hypertension in Pregnancy classification, diagnosis & management recommendations for international practice. Pregnancy Hypertens.

[6] Guideline NICE (2019). Hypertension in Pregnancy: Diagnosis and Management. https://www.nice.org.uk/guidance/ng133.

[7] World Health Organization (2021). WHO Recommendations on Antiplatelet Agents for the Prevention of Pre-Eclampsia.

[8] O’gorman N., Wright D., Syngelaki A., Akolekar R., Wright A., Poon L.C., Nicolaides K.H. (2016). Competing risks model in screening for preeclampsia by maternal factors and biomarkers at 11–13 weeks gestation. Am. J. Obstet. Gynecol..

[9] Tan M.Y., Syngelaki A., Poon L.C., Rolnik D.L., O′Gorman N., Delgado J.L., Akolekar R., Konstantinidou L., Tsavdaridou M., Galeva S. (2018). Screening for pre-eclampsia by maternal factors and biomarkers at 11–13 weeks’ gestation. Ultrasound Obstet. Gynecol..

[10] Tan M.Y., Wright D., Syngelaki A., Akolekar R., Cicero S., Janga D., Singh M., Greco E., Wright A., Maclagan K. (2018). Comparison of diagnostic accuracy of early screening for pre-eclampsia by NICE guidelines and a method combining maternal factors and biomarkers: Results of SPREE. Ultrasound Obstet. Gynecol..

[11] Rolnik D.L., Selvaratnam R.J., Wertaschnigg D., Meagher S., Wallace E., Hyett J., Costa F.d.S., McLennan A. (2021). Routine first trimester combined screening for preterm preeclampsia in Australia: A multicenter clinical implementation cohort study. Int. J. Gynaecol. Obstet..

[12] Tiruneh S.A., Vu T.T.T., Moran L.J., Callander E.J., Allotey J., Thangaratinam S., Rolnik D.L., Teede H.J., Wang R., Enticott J. (2024). Externally validated prediction models for pre-eclampsia: Systematic review and meta-analysis. Ultrasound Obstet. Gynecol..

[13] Bertini A., Salas R., Chabert S., Sobrevia L., Pardo F. (2022). Using Machine Learning to Predict Complications in Pregnancy: A Systematic Review. Front. Bioeng. Biotechnol..

[14] Ranjbar A., Montazeri F., Ghamsari S.R., Mehrnoush V., Roozbeh N., Darsareh F. (2024). Machine learning models for predicting preeclampsia: A systematic review. BMC Pregnancy Childbirth.

[15] Hennessy A., Tran T.H., Sasikumar S.N., Al-Falahi Z. (2024). Machine learning, advanced data analysis, and a role in pregnancy care? How can we help improve preeclampsia outcomes?. Pregnancy Hypertens..

[16] Tiruneh S.A., Vu T.T.T., Rolnik D.L., Teede H.J., Enticott J. (2024). Machine Learning Algorithms Versus Classical Regression Models in Pre-Eclampsia Prediction: A Systematic Review. Curr. Hypertens. Rep..

[17] Zheng D., Hao X., Khan M., Wang L., Li F., Xiang N., Kang F., Hamalainen T., Cong F., Song K. (2022). Comparison of machine learning and logistic regression as predictive models for adverse maternal and neonatal outcomes of preeclampsia: A retrospective study. Front. Cardiovasc. Med..

[18] Sandström A., Snowden J.M., Höijer J., Bottai M., Wikström A.K. (2019). Clinical risk assessment in early pregnancy for preeclampsia in primiparous women: A population based cohort study. PLoS ONE.

[19] Mustafa H.J., Kalafat E., Prasad S., Heydari M.H., Nunge R.N., Khalil A. (2024). Machine learning prediction of hypertension and diabetes in twin pregnancies using characteristics at prenatal care entry: A nationwide study. Ultrasound Obstet. Gynecol..

